# Supplementary material for: A Radiomic Approach for Evaluating Intra-Subgroup Heterogeneity in SHH and Group 4 Pediatric Medulloblastoma: A Preliminary Multi-Institutional Study
Source: Cancers (Basel). 2024 Jun 18;16(12):2248. doi: 10.3390/cancers16122248 (PMC11201623; doi:10.3390/cancers16122248)
Supplement: Supplementary file 1 [file cancers-16-02248-s001.zip › cancers-3057972-supplementary.pdf]

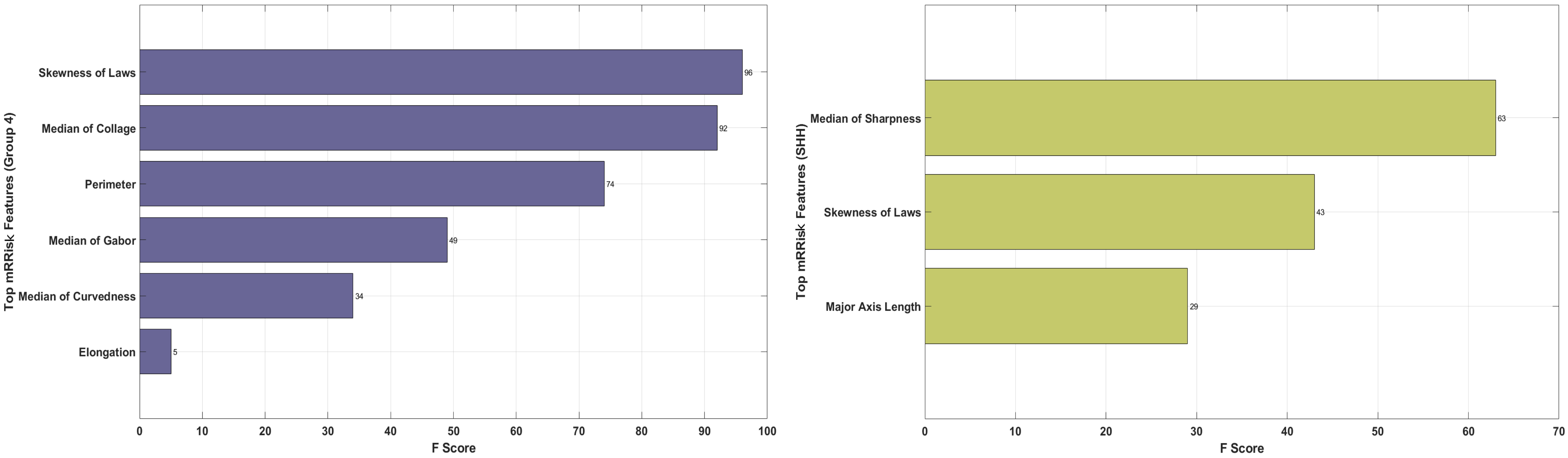

Figure S1: Feature importance graphs for mRRisk descriptor features for both SHH and Group 4 subgroups. The y-axis represents the feature name whereas the x-axis represents the F-score for each feature.

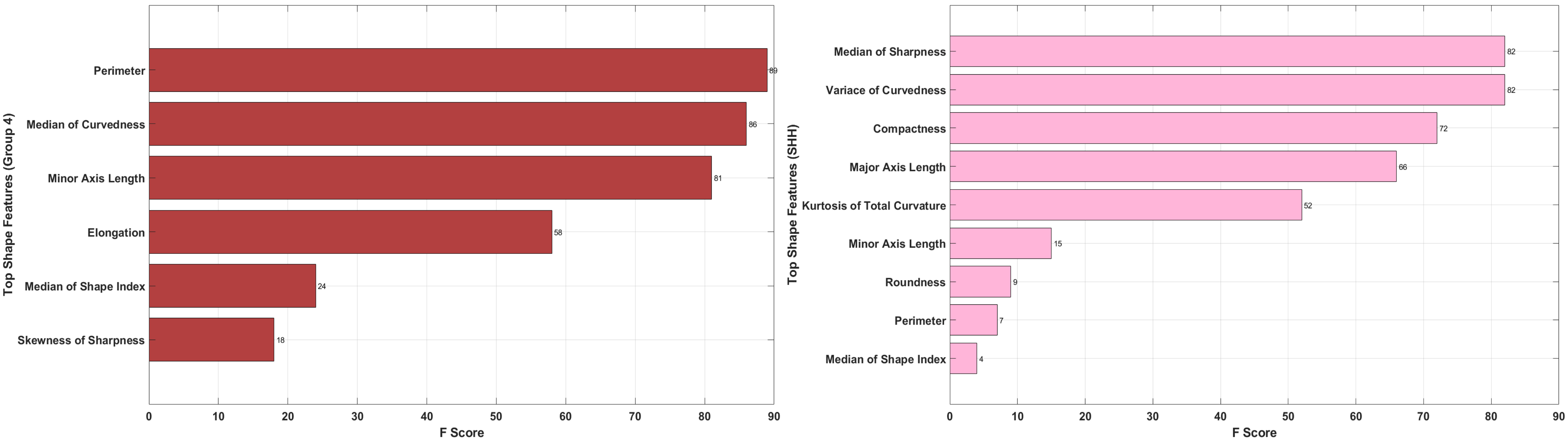

Figure S2: Feature importance graphs for shape features for both SHH and Group 4 subgroups. The y-axis represents the feature name whereas the x-axis represents the F-score for each feature.

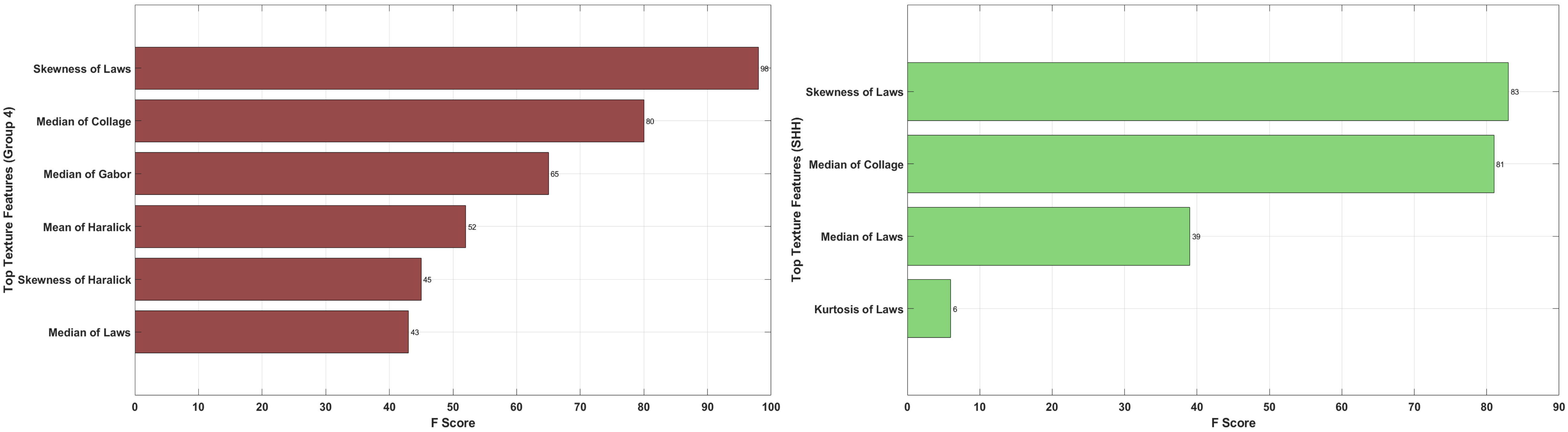

Figure S3: Feature importance graphs for texture features for both SHH and Group 4 subgroups. The y-axis represents the feature name whereas the x-axis represents the F-score for each feature.
